# The Role of Salt Bridges, Charge Density, and Subunit Flexibility in Determining Disassembly Routes of Protein Complexes

**DOI:** 10.1016/j.str.2013.06.004

**Published:** 2013-08-06

**Authors:** Zoe Hall, Helena Hernández, Joseph A. Marsh, Sarah A. Teichmann, Carol V. Robinson

**Affiliations:** 1 Physical and Theoretical Chemistry Laboratory, Department of Chemistry, University of Oxford, Oxford OX1 3QZ, UK; 2 EMBL-European Bioinformatics Institute, Wellcome Trust Genome Campus, Hinxton, Cambridge CB10 1SD, UK; 3 Wellcome Trust Sanger Institute, Wellcome Trust Genome Campus, Hinxton, Cambridge CB10 1SA, UK

## Abstract

Mass spectrometry can be used to characterize multiprotein complexes, defining their subunit stoichiometry and composition following solution disruption and collision-induced dissociation (CID). While CID of protein complexes in the gas phase typically results in the dissociation of unfolded subunits, a second atypical route is possible wherein compact subunits or subcomplexes are ejected without unfolding. Because tertiary structure and subunit interactions may be retained, this is the preferred route for structural investigations. How can we influence which pathway is adopted? By studying properties of a series of homomeric and heteromeric protein complexes and varying their overall charge in solution, we found that low subunit flexibility, higher charge densities, fewer salt bridges, and smaller interfaces are likely to be involved in promoting dissociation routes without unfolding. Manipulating the charge on a protein complex therefore enables us to direct dissociation through structurally informative pathways that mimic those followed in solution.

## Highlights

• Comparison of gas and solution disassembly routes of multiprotein complexes
• Ion mobility-mass spectrometry used to probe structures of dissociation products
• High charge states eject compact monomers and dimers
• Salt bridges and charge density are important for dictating dissociation pathway

## Graphical Abstract

**Figure:**
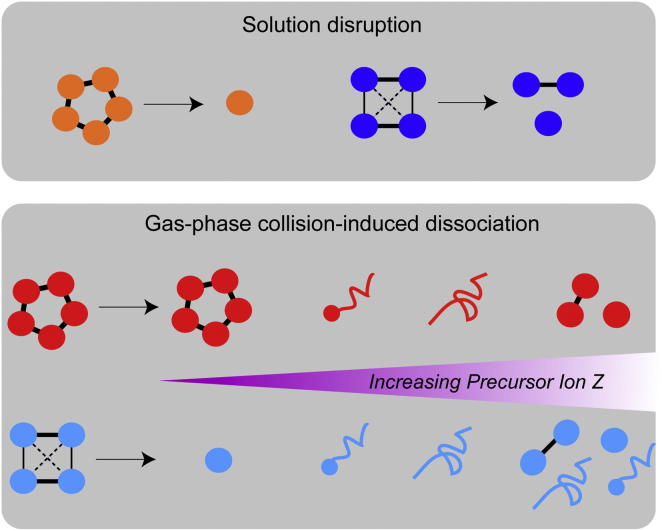

## Teaser

Producing structurally informative gas-phase dissociation pathways for protein complexes is a major goal of structural mass spectrometry. Hall et al. establish correlations between protein structure and dissociation pathways that can be used to predict and control the response to activation in the gas phase.

## Introduction

The assembly of proteins into functional complexes and their characterization is the cornerstone of structural biology. Biophysical tools designed to establish subunit interactions, dynamics, and flexibility of increasingly heterogeneous complexes are continually being developed (Robinson et al., 2007). Mass spectrometry (MS) is one such method that is coming to the fore for defining the subunit composition, connectivity, and topologic properties of protein assemblies (Heck, 2008). Of particular importance have been experiments designed to disrupt large protein assemblies into core modules with use of solution disruption prior to electrospray ionization, which enables subunit interaction maps to be defined (Hernández et al., 2006). Solution-phase disassembly experiments have also been used to support the prediction of any evolutionary pathways of homomeric and heteromeric protein complexes from their X-ray crystal structures (Levy et al., 2008; Marsh et al., 2013). These studies show that, in general, the largest intersubunit interface is formed first and dictates the assembly pattern of the remaining subunits. Conversely, during solution-phase disassembly of the complex the weakest interfaces are broken first. While it is possible to predict these pathways in solution based on relative interface strengths, there is not yet a consensus as to which structural parameters are important for defining the dissociation pathway of a multiprotein complex in the gas phase.

To understand the involved factors, we first have to consider the process of collision-induced dissociation (CID). In the gas phase of the mass spectrometer, following nanoelectrospray ionization, protein complexes are accelerated across a collision cell and subjected to collisions with gas molecules. Because of this process, the internal energy of the ions is increased leading to the Coulombically driven ejection of a highly charged, unfolded monomer (Jurchen and Williams, 2003) and leaving behind a “stripped complex.” We refer to this pathway as “typical” or “dissociation with subunit unfolding.” This typical dissociation results in an asymmetric charge partitioning with respect to mass for protein complexes (Benesch et al., 2006). Although useful for determining the composition of a protein complex and the identity of peripheral subunits, this pathway does not retain the topological information needed for modeling since interactions with neighboring subunits are lost and monomeric subunits unfold. In some cases, however, atypical dissociation has been observed wherein subcomplexes and/or compact monomers are released (van den Heuvel et al., 2006; Boeri Erba et al., 2010). This dissociation pathway therefore preserves the folded state of the ejected subunit, the protein complex, and in some cases neighboring subunit information, and is therefore highly desirable for structural biology applications.

How can we influence which pathway is adopted? Several studies have suggested that the charge state of the complex ion can influence its dissociation pathway (Boeri Erba et al., 2010; Pagel et al., 2010). The charge state distribution observed in mass spectra is dependent on several factors, including solution conditions (Iavarone et al., 2000), interaction with other molecules in the gas phase, and molecular conformation (Mirza et al., 1993). The most common methods to manipulate charge states involve solution additives included in the electrospray solution. Triethylammonium acetate (TEA) and imidazole are often used to lower the charge state because they have higher gas-phase basicities than typical MS buffers (Catalina et al., 2005). Conversely, charge is increased by so-called supercharging reagents used to promote higher charge states than those normally accessible to globular proteins and their complexes. The addition of low volatility reagents with low solution-phase basicity, typically *m*-nitrobenzyl alcohol (*m*-NBA) or sulfolane, is used to cause supercharging (Lomeli et al., 2010). The mechanism for supercharging is still not fully understood but has been attributed to changes in the surface tension of the droplet (Iavarone and Williams, 2003), chemical/thermal denaturation in the final stages of desolvation in the electrospray droplet (Sterling et al., 2012), or direct interaction of the reagent with ionizable groups on the protein (Douglass and Venter, 2012). Whatever the mechanism of this process, the ability to manipulate the charge on a protein complex implies that it may in turn be possible to control the dissociation pathway.

Here, we explore the relationship between the dissociation pathway and the charge state of the protein complex. To do this, we first investigate systematically the effect of charge state on the dissociation of two homomeric complexes, serum amyloid P (SAP) and transthyretin (TTR), and heteromeric tryptophan synthase. We use both charge reduction and supercharging to extend the range of charge states. Using ion mobility-MS (IM-MS), we monitor the collision cross-sections (CCS) of the CID products as a function of the charge state of the complex or ejected subunit. Comparing measured CCS with those calculated for subunits and subcomplexes in their native conformations provides information about the folded state of the dissociation products. Interestingly, SAP and TTR were both found to access atypical dissociation pathways, with the loss of compact monomers and dimers, at higher charge states than available in buffered solution. Tryptophan synthase accessed typical and atypical dissociation routes simultaneously, with both compact and extended forms of the α-monomer ejected in parallel pathways. Extending our study to a further eight complexes and comparing typical and atypical dissociation routes, established here and reported in the literature, we identify a link in which atypical dissociation routes (without subunit unfolding and/or with loss of subcomplexes) tend to be favored by a low number of interfacial salt bridges, small interfaces, and low subunit flexibility. Additionally, a trend was identified wherein complexes with higher overall charge density were found to be associated with atypical dissociation. Importantly this study implies that increasing the charge density of multiprotein complexes enhances the probability of forming structurally informative CID products.

## Results

### How Does the Charge on a Protein Complex Affect Its Dissociation Pathway?

To assess the effects of charge state on dissociation, we first examined two homomeric protein complexes: SAP (5-mer, 125 kDa) and TTR (4-mer, 55 kDa). These complexes represent ideal systems to study because for both proteins gas-phase dissociation routes have been investigated in detail (Sobott et al., 2003; Ruotolo et al., 2007; Beardsley et al., 2009). Moreover, typical CID pathways (loss of unfolded monomeric subunits) as well as atypical pathways (loss of folded monomeric subunits and/or subcomplexes) have been observed for charge-reduced TTR (Pagel et al., 2010) and supercharged SAP (Hall et al., 2012a).

**Figure 1:**
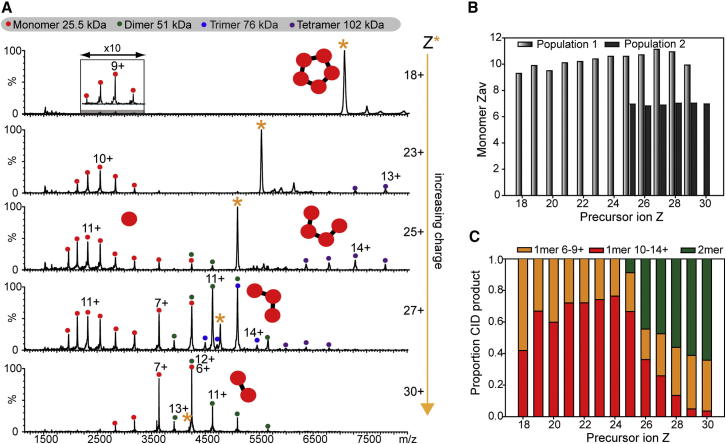
CID of Serum Amyloid P 5-mer (A) MS/MS of SAP 5-mer with increasing precursor ion charge state. MS/MS spectra were acquired at the same laboratory frame energy (collision energy × Z) with the exception of the lowest charge state (18+), where higher energies were required in order to effect dissociation. (B) Average charge state (Z_*av*_) of the dissociated monomer was plotted against the precursor ion charge state. For Z ≥ 25+, two populations of monomer are observed. (C) The relative intensities of monomer and dimer CID products in the MS/MS spectra are plotted against precursor ion charge state. Precursor charge states were generated as follows: to 200 mM AA: 18–21+ (10 mM TEA), 22–25+ (no additive), and 26–30+ (1% *m*-NBA). See also Figure S1.

First, we investigated the dissociation of SAP making use of charge reduction with TEA and supercharging using *m*-NBA. A wide range of charge states was generated (18+ to 30+) and different precursor ions were selected in the quadrupole for tandem MS (MS/MS) (Figure 1A). The lowest charge states (18+) were highly stable and resistant to gas-phase dissociation, with low intensity backbone cleavage products and monomeric subunits observed. However, higher charge states (23+ to 30+) readily dissociated via loss of monomers. Considering the dissociation of 25+ ions for pentameric SAP, the most intense peaks corresponding to the dissociated monomers (25.5 kDa), and the stripped tetrameric complexes (102 kDa) carry 11+ and 14+ charges, respectively. The fact that the dissociated monomer carries almost half the total charge of the intact complex, but corresponds to only one fifth of the mass is consistent with the established asymmetric distribution of charge with respect to mass observed in typical CID pathways (Jurchen and Williams, 2003). Interestingly, as the charge state increases, through the addition of supercharging reagent, loss of low-charged monomers and dimers was observed.

To investigate the dependence of the charge of the complex ion on the dissociation products formed, we plotted the average charge state of the dissociated monomer (Z_*av*_) against the precursor ion charge state (Z) (Figure 1B). From 18 to 24+, as the precursor ion charge state increases, Z_*av*_ for the monomer also increases. For Z > 24+, two distinct populations of monomer are observed, the emerging population having significantly lower charge states. Moreover, as the precursor ion charge state increases from 25+ to 30+, the high charge monomer population decreases in intensity relative to that of the low-charge monomer (Figure 1C). Increasing proportions of dimer were also observed with an increase in precursor ion charge (Z > 24+). These observations suggest that for Z ≥ 25+, a second dissociation pathway becomes accessible in which low-charged monomers and dimers are ejected. The charge state distributions are consistent with the loss of two dimers and one monomer, or the loss of dimer and trimer, which subsequently splits into dimer and monomer. In either scenario, increasing the precursor ion charge state of SAP results in this alternate and more structurally informative pathway being favored.

**Figure 2:**
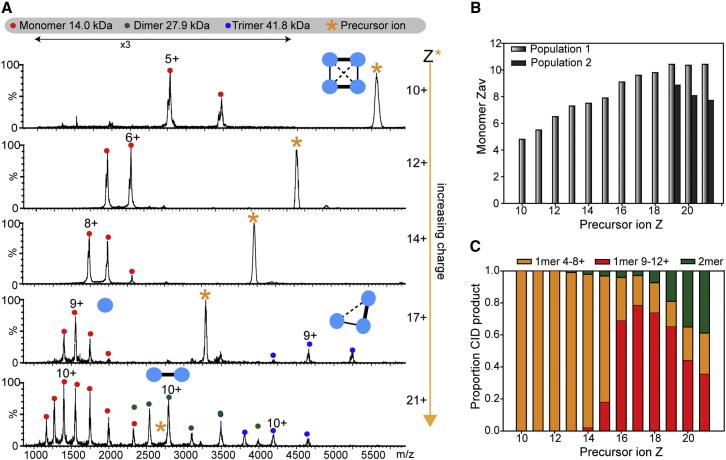
CID of Transthyretin (A) MS/MS of TTR with increasing precursor ion charge state. Initially, the dissociated monomer charge state increases with the precursor ion charge state. Supercharged TTR (21+) can dissociate via alternate pathways, losing lower charged monomers and dimers. (B) Z_*av*_ of the dissociated monomer is plotted against precursor ion charge state. At Z ≥ 19+, two distinct populations of monomer are observed. (C) Relative intensities of monomer and dimer CID products are plotted against precursor ion charge state. Precursor charge states were generated as follows: to 200 mM AA: 10–13+ (20 mM DBU), 14–16+ (no additive), and 17–21+ (1% *m*-NBA). See also Figure S2.

How widely applicable is this approach? Using the same charge manipulation protocols as for SAP above, the dissociation of TTR was studied (Figure 2A). Similar to SAP, the lowest charge states (10+) were highly stable and ejected peptides and low-charged monomers, as reported previously (Pagel et al., 2010). Increasing the charge state (12+ to 14+) caused an increase in the average charge state of the dissociated monomer from 6.5+ to 7.5+ (Figure 2B). In general, the ejected monomer removed approximately half the number of charges present on the precursor ion. For Z ≥ 19+ two populations of monomer were observed, with distinct charge state distributions. Furthermore, for Z > 17+ increasing populations of dimer were observed with an increase in precursor ion charge state. The dimer could arise from direct dissociation of the tetramer into two dimers or from the sequential loss of two monomers. Considering the charge state of the TTR tetramer (21+), if a dimer (Z_*av*_ ∼10+) were to form from the sequential loss of two monomers from the tetramer, the sum of charges on the two ejected monomers would have to be 11+. However, the dissociated monomers have charge states from 6+ to 12+ and are thus too highly charged to sum to 11+. The low intensity peaks for charge states 7+ and 8+ of the dimer could arise from the sequential loss of two monomers, and therefore we tentatively assign these charge states to the stripped complex. However, based on charge state, we conclude that a major population of dimer is expelled intact from the tetrameric complex.

This atypical dissociation pathway, resulting in the ejection of dimers and low-charge monomers was observed for high charge states of both SAP and TTR, accessed by the addition of supercharging reagents. There are two possible explanations for this behavior: either the high charge state of the precursor ion dictates the atypical behavior, or the supercharging reagent itself affects the complex by weakening subunit interactions. To test these two possible scenarios, the dissociation of SAP 26+ was examined following electrospray from either ammonium acetate (AA) or *m*-NBA containing solutions (Figure S1 available online). The MS/MS spectra for the 26+ ions are closely similar under identical instrument parameters, regardless of whether the ion resulted from supercharging with *m*-NBA or was electrosprayed directly from AA buffer. This implies that in this case it is the charge state, rather than the presence of solution additive, that governs the dissociation route.

### Gas-Phase versus Solution-Phase Disassembly

**Figure 3:**
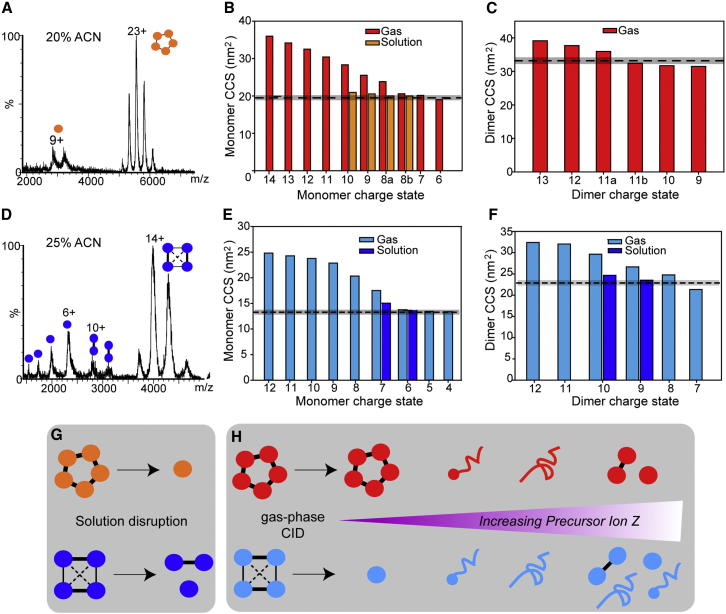
Comparison of Gas Phase Dissociation and Solution Disassembly (A–H) SAP (orange/red) and TTR (cyan/blue) were disrupted in solution by the addition of acetonitrile (A) and (D). CCS were measured for the monomer formed in solution and compared with CCS of monomer resulting from gas phase dissociation (B and E), and values were calculated from the X-ray crystal structure (dashed line with ± 3% error shaded). CCS were also measured for dimer formed in solution, gas phase, and calculated from crystal structure (C and F). Dimer was not formed in solution for SAP. CCSs for each charge state (gas phase products) are the average value calculated from multiple MS/MS experiments of different precursor ions that give the same charge product ions (SD < 1%–2%). Multiple conformations are indicated by *a* or *b* for SAP monomer (8+) and dimer (11+). Schematics show solution disruption (G) and CID, with increasing precursor ion Z (H). See also Figure S3.

To compare these gas-phase dissociation pathways with those in solution, SAP and TTR were partially disrupted by addition of organic solvent to form subcomplexes (Hernández et al., 2006). Upon the addition of acetonitrile, SAP formed monomers, while TTR formed both monomers and dimers (Figures 3A and 3D). To obtain insights into the structures of these solution dissociation products, we used IM-MS. IM starts with injection of an ion packet into a mobility cell filled with inert neutral gas. Aided by a weak electric field, the ions travel through the cell, colliding with neutral gas molecules in their path. Ions with a compact globular shape undergo fewer collisions and thus possess higher mobility than ions with more extended conformations enabling their separation. Drift times measured in IM are usually converted into CCS values that are independent of specific instrument parameters (Wyttenbach and Bowers, 2011). The CCS for the solution disassembly products for these two homomers were therefore measured and compared with CCS calculated from the corresponding X-ray crystal structures. Close agreement was found between the solution-phase disassembly products of TTR and SAP and the analogous structures calculated from the Protein Data Bank (PDB). This is consistent with previous studies in which the CCS of solvent-disrupted subunits and subcomplexes were unaffected relative to those calculated for the corresponding species in their native states (Pukala et al., 2009; Hall et al., 2012b).

The solution-phase disassembly products were then compared with those generated in the gas phase. Solution-formed monomers of SAP (charge states 8–10+) were in good agreement (<5% deviation) with the CCS of the lowest charge states (6–7+) of the gas-phase dissociated monomer (Figure 3B). Gas-phase monomers (8+) consistently exhibited two conformations, irrespective of the precursor ion charge state, one similarly compact to the solution monomer (8+[*b*]) and one unfolded (8+[*a*]). Because no dimer was formed in solution, the gas-phase dimer was compared with that of a dimer formed by subtraction of subunits from the X-ray structure (Figure 3C). Two conformations were evident for the gas phase dimer (11+): compact (11+[*b*]) and slightly unfolded (11+[*a*]). The compact structure has a CCS within 3% of that calculated from the X-ray structure. Higher charge states (12+ and 13+) showed some unfolding, while CCS of the low-charge dimers (9+ and 10+) are within 4%–5% of the calculated CCS. We conclude that the low-charge dimers and monomers, formed in the gas phase, have compact structures in line with CCS predicted from X-ray structures.

Similarly, CCS were measured for the monomer (6–7+) and dimer (9–10+) of TTR formed in solution, and compared with CCS of the corresponding gas phase products and with values calculated from the X-ray crystal structure (Figures 3E and 3F). Two dimers are possible for TTR: the calculated CCS was based on the dimer formed by breaking the least amount of interface (Marsh et al., 2013). Gas phase monomers (4–6+) were in good agreement with both the CCS of the solution-disrupted monomer and the X-ray crystal structure (<2% deviation). The CCS for the lowest charge state of the gas phase dimer (7+) was 5% smaller than that expected from the X-ray crystal structure. Conversely, higher charge states had CCS greater than anticipated, consistent with unfolding. Interestingly, low charge states of the gas phase monomer could be accessed by dissociation of either charge-reduced or supercharged TTR. The CCS for these low-charge monomers, however, is indistinguishable within the precision of the measurement (Figure S2). We conclude therefore that the CCS of the gas phase dissociation products for the complexes described above are independent of the charge state of the precursor ion from which they are ejected.

The stripped complexes formed in the gas phase during dissociation experiments (SAP 3-mer, SAP 4-mer, and the TTR 3-mer) were also measured using IM-MS. Experimental values were compared with CCS calculated for the corresponding X-ray structures, formed by removing monomeric subunits to form “clipped” X-ray structures. The CCS for the lowest charge states of both SAP and TTR trimers were in good agreement (<5% deviation) with calculated CCS (Figure S3). However, rearrangement to more stable structures, with similar CCS to the clipped X-ray crystal structures cannot be ruled out. In contrast, the lowest charge states for the SAP 4-mer have significantly smaller CCS (∼15%) than expected from the crystal structure. This suggests substantial collapse is occurring in the gas phase, burying the newly exposed surface area resulting from dissociation of subunits (Zhou et al., 2013). These results are in line with previous studies in which stripped complexes, particularly those whose subunits are arranged in ring-like architectures, have been shown to undergo significant collapse following dissociation in the gas phase (Pukala et al., 2009; Knapman et al., 2010; Zhou et al., 2013). As with the monomers and dimers examined above, CCS of the SAP trimers and tetramers and TTR trimers increase with charge state, consistent with the unfolding of one or more monomers.

### Dissociation of Heteromeric Tryptophan Synthase

**Figure 4:**
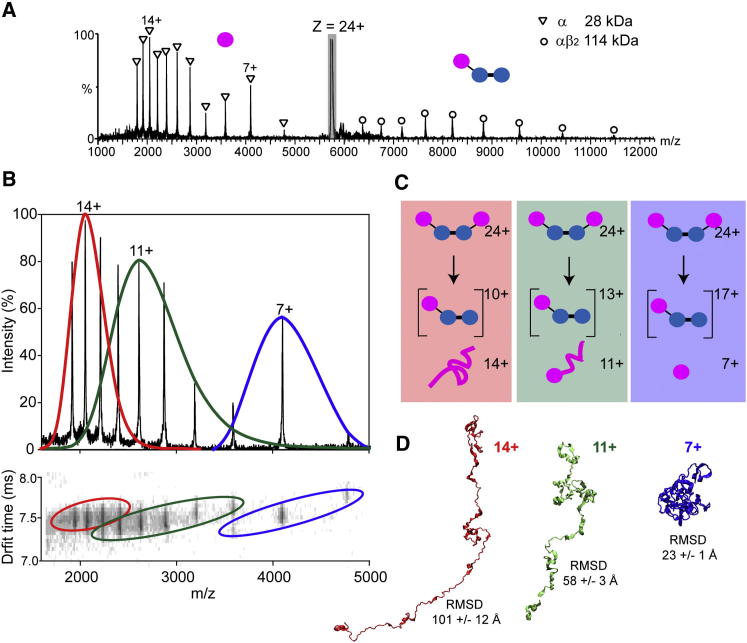
Gas Phase Dissociation of Tryptophan Synthase (A) MS/MS of tryptophan synthase (24+) reveals loss of α subunit. (B) Three distinct populations of ejected α subunit are observed and confirmed via a drift time versus *m/z* contour plot. (C) Schematic for the proposed parallel routes for gas phase dissociation: loss of high, intermediate, and low-charge subunits (red, green, and blue). (D) MD simulations of tryptophan synthase (24+), over a linear temperature gradient, recapitulates the ejection of an α-monomer with different degrees of compactness. Charges assigned to the α subunit were 14+, 11+, and 7+, as determined experimentally. Residual charges were evenly distributed over the accessible basic residues on the remaining three subunits. The rmsd of the dissociated α subunit from that bound in the native structure is shown. See also Figure S4.

Having demonstrated that SAP and TTR can access dissociation pathways that result in compact monomers and dimers with CCS similar to those formed during solution disassembly, the next step was to see if this could be achieved for a heteromeric complex. Tryptophan synthase (143 kDa) with its established solution disassembly was chosen (Hall et al., 2012b). First, the CCS for a wide range of charge states of the intact complex were measured (Figure S4). This included those formed via charge reduction with TEA (19–22+), from AA-buffered solutions (22–27+) and those generated via supercharging with *m*-NBA (27–31+). These CCS were compared with those reported previously for the same complex in AA-buffered solution and were within error (Hall et al., 2012b). However, charge states 30+ and 31+ had larger CCS than anticipated, consistent with unfolding. MS/MS experiments of the 24+ ion of tryptophan synthase in AA buffer resulted in loss of α-monomers (Figure 4A) with charge states 6+ to 17+. Closer inspection of MS/MS and IM data revealed three distinct populations of α-monomers, with overlapping Gaussian charge state distributions centered on 14+, 11+, and 7+ (Figure 4B). Examination of the arrival time distributions for the precursor ion showed no evidence for multiple conformations, suggesting that there are three parallel dissociation routes resulting in α subunits with three different charge state distributions (Figure 4C).

To explore the possible structure of these three different α subunits, we used solvent-free MD simulations to mimic CID, using a temperature gradient to impart increasing amounts of energy to the system (Hall et al., 2012a). Three 1 ns simulations were carried out, differing only in the initial distribution of charges over the complex. The experimentally determined charge states of the monomeric subunit populations, observed in MS/MS experiments of tryptophan synthase 24+, were used to assign 14+, 11+, or 7+ charges to the α subunit. The residual charges were distributed evenly over the accessible basic residues of the remaining three subunits. During these simulations, dissociation of the α-monomer (14+, 11+, or 7+) was observed. Representative structures are shown (Figure 4D). The extent of unfolding was directly related to the number of charges originally assigned to the dissociating monomer. Charge distributions for the intact complex, which were more symmetric with respect to mass, i.e. 7+ on one α-monomer, and 17+ distributed over the αββ trimer, resulted in the dissociation of compact α-monomer. In contrast, the most asymmetric charge distribution, 14+ (α subunit) and 10+ (αββ trimer), gave rise to the dissociation of an extended α subunit. The dissociated subunit (7+, 11+, or 14+) had radii of gyration of 24 (±3), 65 (±6), and 118 (±12) Å, respectively, indicating progressive unfolding with charge. For comparison, the radius of gyration of the α-monomer in a random coil state is estimated to be ∼54 Å (Kohn et al., 2004). The most populated conformations for the ejected α-monomer therefore are consistent with compact (7+), partially unfolded (11+), and highly unfolded (14+) α subunits.

**Figure 5:**
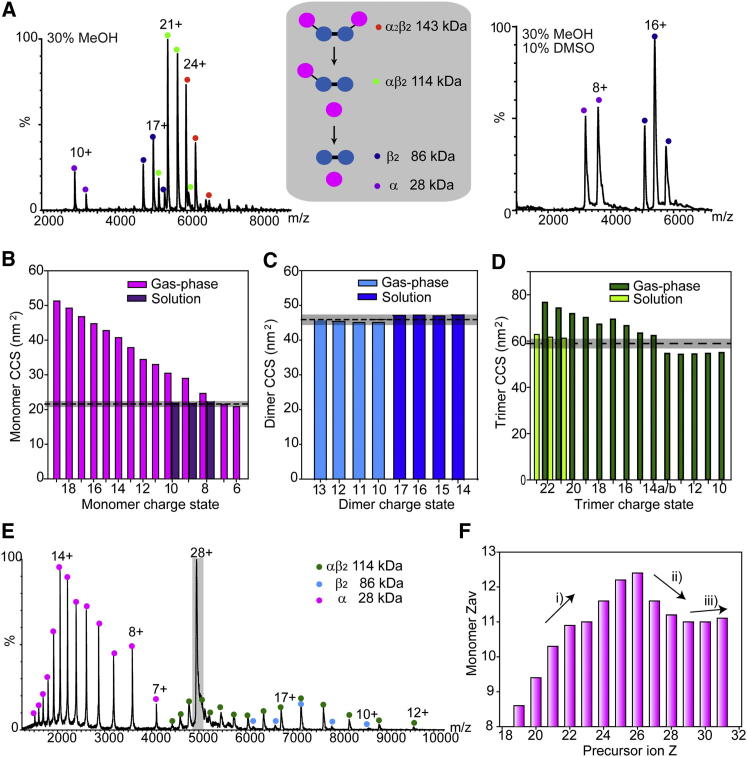
Comparison of Tryptophan Synthase Gas and Solution Phase Disassembly (A) Tryptophan synthase dissembles in solution by the loss of α-monomers to give αβ_2_-trimer, β_2_-dimer and α−monomer, depending on solution conditions. (B–D) CCS were measured for the α-monomer (B), β_2_-dimer (C), and αβ_2_-trimer (D) formed in solution (purple, blue, pale green, respectively), and compared with CCS of the corresponding gas phase product (pink, cyan, green). Two conformations of the gas phase trimer 14+ are identified (*a* and *b*). (E) At higher collision energy (28+) two monomers dissociate, allowing CCS measurements of the β_2_-dimer. (F) Z_*av*_ for the dissociated α-monomer is plotted against precursor ion charge state. Charge states were generated as follows: to 200 mM AA: 19–21+ (20 mM TEA), 22–26+ (no additive), and 27–31+ (1% *m*-NBA). See also Figure S5.

These gas phase disassembly routes were then compared with those generated in solution (Figure 5). Tryptophan synthase disassembles in solution to give αβ_2_-trimer, β_2_-dimer, and α-monomer, depending on the composition of the solution: 30% methanol (Figure 5A, LHS) or 30% methanol/10% DMSO (Figure 5A, RHS). CCS were measured for the α-monomer, β_2_-dimer, and αβ_2_-trimer formed in solution and compared with CCS of the corresponding gas phase product (Figures 5B–5D). CCS of the low-charge states of the monomer (6+ and 7+) compared well with the CCS of the α subunit generated in solution (8–10+) and with the value calculated from the X-ray structure (<5% deviation). During gas phase experiments, the loss of two α-monomers was observed at higher laboratory frame energies (Figure 5E), and therefore CCS was measured for the β_2_-dimer in addition to the α-monomer and αβ_2_-trimer. The β_2_-dimer generated in the gas phase and in solution has CCS within 4% of that calculated from the X-ray structure. For the αβ_2_ trimer, solution phase disassembly matches well with the value calculated from the PDB (within 2%–7%). Two conformations were observed for the 14+ gas phase αβ_2_ trimer, one with evidence for collapse (14+[*b*]) and one unfolded (14+[*a*]). Charge states of the gas phase trimer above and below 14+ similarly are unfolded and collapsed, respectively.

To explore the relationship between the charge state of the protein complex and the charge on the ejected subunit, MS/MS was performed (Figure S5A). The change in relative intensities of the charge state distributions of the α subunit, defined as low (6–9+), intermediate (10–13+), and high (14–17+) were plotted as a function of the charge state of the complex (precursor ion Z; Figure S5B). Interestingly, the maximum population of the highest charged ejected monomer occurs not from the highest precursor ion charge state as might be anticipated, but from an intermediate charge state of 25+. Plotting the Z_*av*_ of the dissociated α-monomer against the precursor ion charge state (Figure 5F) reveals three distinct trends: (1) initially as the precursor ion charge state increases, the Z_*av*_ of the monomer increases. This corresponds to a reduction in intensities of the lowest charge states and an increase in the higher charge states. (2) For Z > 26+, a corresponding decrease in monomer Z_*av*_ is observed. This is consistent with an increase in tryptophan synthase charge favoring the dissociation of lower-charge monomers. This shift in trend is analogous to observations made for supercharged TTR and SAP wherein high charge states resulted in ejection of compact low-charge monomers and dimers. (3) For Z > 29+, a slight increase in monomer Z_*av*_ is observed with increasing precursor ion charge state. This observation can be explained because these ions (30+ and 31+) are partly unfolded prior to MS/MS.

Overall, our experiments on these three complexes have shown that dissociation of high charge states can deliver compact higher-order oligomers (dimers for SAP and TTR) with CCS that closely match the corresponding species generated following solution phase disruption and CCS calculated from atomic coordinates. Similarly, low charge states of the individual subunits agree closely with CCS measured in solution and calculated from X-ray structures. Higher charge states of individual subunits populate extended forms consistent with significant unfolding. Fragmentation of subunits and/or further dissociation of oligomers by triple quadrupole MS or higher analyses has the potential to reveal additional structural information (Huang and McLuckey, 2010). Importantly, however, we have shown that it is possible to recapitulate solution disassembly pathways when sufficient charge is added to the intact complexes.

### Factors Directing Gas Phase Dissociation Pathways

**Figure 6:**
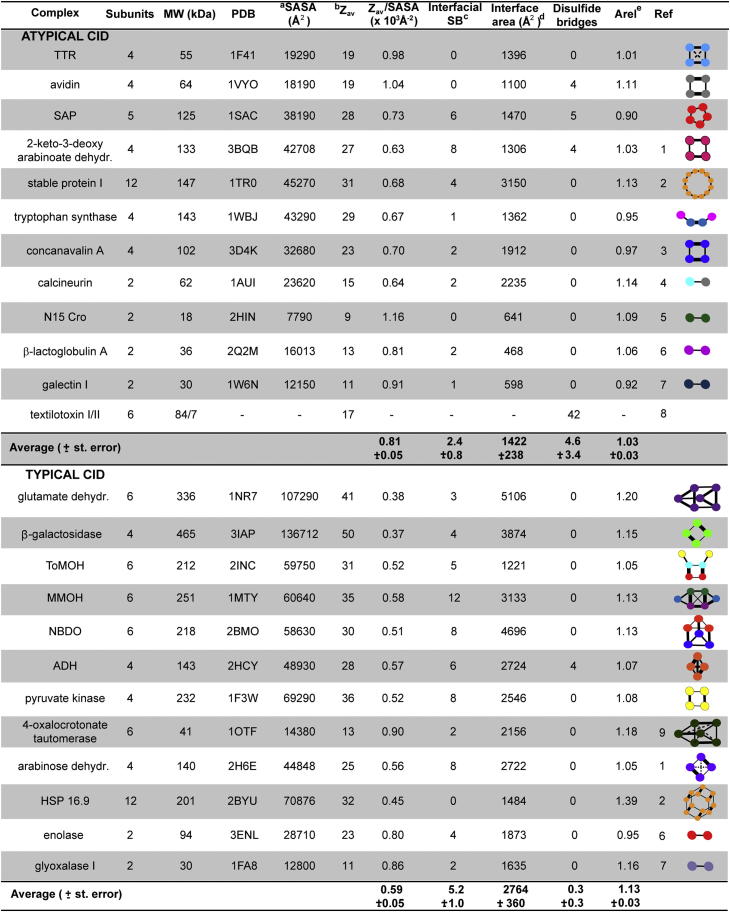
Properties of a Range of Multiprotein Complexes and Their Dissociation Pathways Complexes that dissociate atypically and typically are shown in the upper and lower parts of the table, respectively. ^a^SASA was calculated using PISA. ^b^Z_*av*_ is the average charge state observed in 1% *m*-NBA (this study) or reported under varying solution conditions. ^c^Minimal number of interfacial salt bridges broken during the first step of in silico disassembly based on breaking the minimum interfacial surface area. Salt bridges were calculated using PISA (Krissinel and Henrick, 2007). ^d^Interface area broken during first step of in silico disassembly. ^e^*A*_rel_ is the subunit relative SASA, and a proxy for flexibility. References: 1 (van den Heuvel et al., 2006); 2 (Boeri Erba et al., 2010); 3 (Zhou et al., 2013); 4 (Kükrer et al., 2012); 5 (Dodds et al., 2011); 6 (Loo, 2001); 7 (Versluis et al., 2001); 8 (Aquilina, 2009); and 9 (Fitzgerald et al., 1996). See also Figure S6 and Table S1.

Given the success in manipulating the charge state to cause different CID pathways for the three complexes examined above, we investigated the general applicability by extending our study to eight additional protein complexes (Figure 6; Figure S6). These comprised avidin, alcohol dehydrogenase (ADH), pyruvate kinase, glutamate dehydrogenase, methane monooxygenase hydroxylase (MMOH), toluene/*o*-xylene monooxygenase hydroxylase (ToMOH), nitrobenzene dioxygenase (NBDO), and β-galactosidase. Interestingly, the atypical pathways observed for SAP and TTR were also observed for avidin (Figure S7; Table S1). The remaining seven complexes dissociated exclusively through the more typical dissociation pathway, with loss of highly charged unfolded subunits even from the highest charge states accessed by supercharging reagents. In the case of β-galactosidase a highly charged subunit fragment was lost. These observations suggest that not all complexes are capable of undergoing atypical dissociation despite accessing high charge states. This prompts the question as to why high charge states of certain protein complexes can access alternate dissociation pathways, while others are unable to expel folded subunits or higher oligomers.

To explore a link between the structure of the protein complex and the dissociation pathway, we considered several options including the flexibility of the subunit, the average charge density, and the interfacial interactions. To increase the data set, we also included 13 complexes previously reported as undergoing typical or atypical dissociation (Figure 6), extending our definition of atypical dissociation to include symmetric charge partitioning in dimer dissociation.

We considered the role of subunit flexibility: the atypical dissociation of homomeric textilotoxin (Aquilina, 2009) has been attributed to the high proportion of disulfides (seven disulfides per subunit), which inhibit unfolding and promote dissociation of compact species. However, the remaining complexes undergoing atypical CID had only 0–1 disulfide bridges per subunit, suggesting that this property cannot account for atypical dissociation in all cases (Figure 6). A second measure of subunit flexibility is the relative solvent-accessible surface area (SASA) parameter (*A*_rel_), which describes the ratio between observed and predicted SASA for a protein of given molecular mass. *A*_rel_ values calculated for both monomers and bound protein complex subunits were previously demonstrated to show strong correlations with binding-induced conformational changes and intrinsic flexibility (Marsh and Teichmann, 2011). Interestingly, analysis of the proteins under study here reveals a significant tendency for atypically dissociating complexes to have subunits with lower *A*_rel_ values than typically dissociating complexes (1.03 versus 1.13, p = 0.011; Figure 6). This suggests that proteins that undergo atypical dissociation without unfolding also tend to be considerably more compact and less flexible in their unbound states. In contrast, proteins that undergo typical dissociation are generally more flexible and thus unfold more readily.

Next, we studied the role of charge on the complexes reported to undergo atypical CID. High charge states of a cytochrome *c* dimer were shown to dissociate similarly to those dimers whose subunits had been conformational restricted by crosslinking (Jurchen and Williams, 2003). This suggests that the charge on the precursor ion of the complex is important. This was further explored in a study of stable protein 1, in which it was suggested that in addition to structural features restricting unfolding, a high charge density contributed to the unusual dissociation of this complex (Boeri Erba et al., 2010). This prompted us to consider the charge per unit area for Z_*av*_ in MS (Figure 6). Complexes for which atypical dissociation pathways were observed had significantly higher gas phase charge densities on average (0.81 × 10^−3^ versus 0.59 × 10^−3^ Å^−2^, p = 0.003), than the complexes dissociating typically. Therefore, this implies that charge density is an important determinant of dissociation without unfolding.

**Figure 7:**
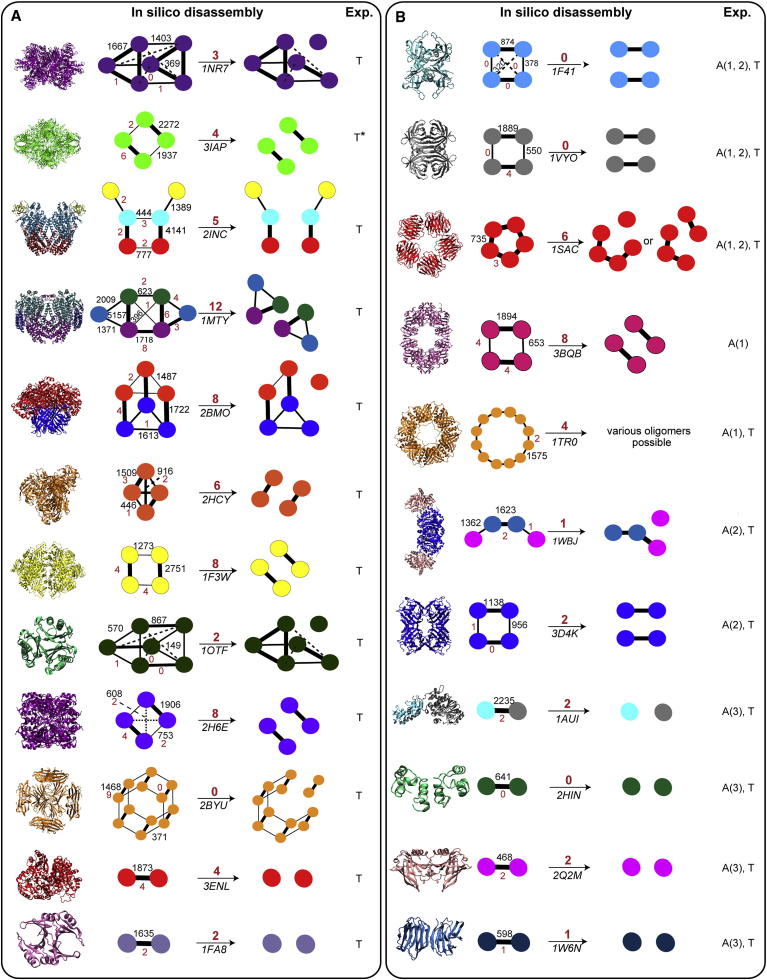
In Silico Disassembly of Complexes Using the Interface Model In silico disassembly proceeds by the route in which the least amount of interface area is broken. The crystal structures of the complexes undergoing typical (A) or atypical CID (B) are shown with their corresponding PDB numbers, and ball-and-stick representations (ball, monomer; stick, interface). Interface areas in Å^2^ (black) and number of salt bridges (red) are marked. Interfacial salt bridges broken in each in silico disassembly step are given above the reaction arrows. Gas phase disassembly routes are defined as typical dissociation of an unfolded monomer (T) or fragment (T^∗^). Atypical dissociation is represented as follows: formation of subcomplexes A(1), loss of compact monomers A(2), and symmetric charge partitioning between dissociation products A(3). See also Figure S7.

Finally we examined the interfaces, specifically the number of interfacial salt bridges, because electrostatic interactions increase in strength in the gas phase (Robinson et al., 1996) and have been implicated in stabilizing protein structure in vacuo (Breuker et al., 2011). The number of interfacial salt bridges broken during dissociation could therefore influence the likelihood of dissociation without prior unfolding, i.e., “direct dissociation” into subcomplexes and/or subunits. To test this, we considered the most likely route for direct dissociation without unfolding by making use of a simple predictive model that assumes that the strength of each interface is proportional to the surface area buried. Consequently, the smallest interfaces will be broken first during in vitro disassembly (Figure 7; Levy et al., 2008; Marsh et al., 2013). These predicted pathways represent the most likely disassembly route during solution disruption and therefore during atypical CID, which tends to recapitulate solution disassembly. The number of salt bridges that would be broken during the first disassembly step in solution was calculated. Interestingly the complexes that dissociate atypically tend to be those in which the fewest salt bridges need to be broken (Figures 6 and 7) during in silico disassembly (2.4 versus 5.2, p = 0.018). This suggests that a higher number of salt bridges increase the energy barrier to dissociate without unfolding (see also Supplemental Discussion). This is in accord with previous reports that showed that dimers with fewer electrostatic interactions between subunits were more likely to undergo symmetrical charge partitioning upon dissociation (Loo, 2001; Dodds et al., 2011).

A trend is also apparent for size (as measured by the SASA or molecular weight) of the intact complex and its preferred dissociation route (see also Supplemental Discussion). Complexes that dissociate atypically are generally smaller than those dissociating typically, even when the dimeric complexes are excluded. The size of the complex is correlated with the area of the interfaces. Comparing the amount of interface broken between the two groups during the first in silico disassembly step (Figure 6) reveals a significant tendency for complexes undergoing atypical dissociation to dissociate by breaking a smaller interface area (1,422 versus 2,764 Å^2^, p = 0.003). Therefore dimers, which only have one interface to break, and other low-molecular-weight complexes are more likely to undergo atypical CID than high-molecular-weight complexes with larger, multiple interfaces. This might suggest that the salt bridge trend is also related to the size of the complex, presuming that low-molecular-weight complexes with smaller interfaces have fewer interfacial salt bridges. However, further examination of the relationship between salt bridges and size shows that the number of interfacial salt bridges is poorly correlated to both complex size (*R*^*2*^ = 0.2), and interface size (*R*^*2*^ = 0.2). Furthermore there is no significant difference in the interfacial salt bridge density (i.e., normalized over interface area) between the atypical and typical dissociation groups (p = 0.3). These observations indicate the interfacial salt bridge trend is unrelated to the complex size. On the other hand, the charge density is strongly related to the size of the complex, with charge density increasing exponentially with decreasing molecular weight (see Supplemental Discussion). Overall, therefore, small complexes have a tendency to be more susceptible to atypical CID than larger complexes due to both smaller interfaces and higher charge densities.

## Discussion

It is interesting to compare the atypical CID pathways observed for supercharged protein complexes with surface-induced dissociation (SID). While the timescale of CID is on the order of microseconds, SID in which dissociation results from the protein complex colliding with a solid surface occurs much faster, on the picosecond time scale. The rapid deposition of energy in SID results in dissociation occurring on a faster time scale than unfolding, and as a result, dissociation tends to occur in a charge-symmetric fashion (Beardsley et al., 2009). SID often results in subcomplex dissociation, rather than the loss of single monomers as typically observed in CID (Wysocki et al., 2008). These products have been shown by recent IM experiments to be compact, with CCS in good agreement with calculated values (Zhou et al., 2012). Although IM-MS cannot report on the precise packing of secondary structural elements or of side chain rearrangements, the available experimental evidence implies that a compact state of a protein can survive following gas phase dissociation, suggesting that these products are native-like. The results presented here show that, similar to SID, subcomplexes and compact monomers can be observed following CID of supercharged species. Further comparison of dissociation techniques, for example that achieved by blackbody infrared radiative dissociation (BIRD) could reveal further insights into the atypical dissociation phenomenon (Felitsyn et al., 2001; Sinelnikov et al., 2007).

Dissociation and unfolding are often considered as competing processes. Typically, dissociation of multimeric complexes is achieved at higher energies than required for unfolding. Consequently, CID proceeds via the unfolding of a single subunit, which is ejected when energy input is sufficient. Factors that inhibit unfolding or promote dissociation will drive complexes toward atypical CID pathways, where subunits or subcomplexes are ejected without unfolding. Therefore, low subunit flexibility, for example in complexes with many disulfides or with low subunit *A*_rel_ values, will result in inhibition of unfolding, with atypical CID predominating. Breaking smaller interfaces and fewer salt bridges during disassembly lowers the barrier for dissociation and consequently promotes atypical CID without unfolding. Intuitively complexes with high charge densities could be considered more susceptible to both unfold and to dissociate as a consequence of their greater intra- and intermolecular Coulomb repulsion. What therefore renders dissociation more favorable over unfolding at very high charge densities?

To understand the propensity for unfolding versus dissociation, we considered the relationship between the dissociation route of tryptophan synthase and the distribution of charges over the complex in MD simulations. Compact α-monomers for tryptophan synthase were observed following MD simulations in which the precursor ion had a symmetric charge distribution (Figure 4D). In contrast, an initial asymmetric charge distribution resulted in dissociation of the highly charged α-monomer, which was unfolded. The more asymmetric the initial charge distribution, the more unfolded the dissociated monomer. This is consistent with previous theoretical calculations where perfect symmetric charge partitioning in multimeric complexes resulted in the largest intermolecular repulsion, lowering the barrier for dissociation. Asymmetric charge partitioning, on the other hand, lowers the barrier for unfolding through increased intramolecular repulsion, with the balance between inter- and intramolecular Coulomb energy controlling the dissociation path of complexes (Wanasundara and Thachuk, 2007, 2009). This implies that charge partitioning plays a major role. It is tempting to speculate that charge migration, which occurs concomitantly with unfolding, may be slower for precursor ions with higher charge states, and fewer free basic residues.

In summary, while many structural features may play a role in directing dissociation of protein complexes, we have identified and explored several important factors here. The key determinants for retaining structure in the gas phase appear to be subunit flexibility, interface size, and electrostatic nature, and the ability to attain high charge densities.

### Conclusions

Dissociation of complexes into their component subcomplexes enables information on subunit connectivity and, when coupled with ion mobility, provides topological restraints for modeling. A major goal in these dissociation experiments therefore is to maintain complexes and their products in folded conformations whenever possible. While this is generally straightforward in solution disruption experiments, in the gas phase, unfolding prior to dissociation is a preferred route. However, a second less common gas phase dissociation pathway, in which compact monomers and/or dimers are ejected, is preferable from a structural viewpoint. We investigated the factors that influence the dissociation pathway by selecting 11 homomeric and heteromeric complexes and supplementing these data with examples from the literature. We find that by increasing the overall charge density, for a subset of these complexes, we can drive dissociation along the second preferred pathway and consequently generate gas phase products analogous to those formed in solution. CCS for these compact gas-phase dissociation products are in good agreement with the corresponding solution disassembly products and with those calculated from X-ray crystal structures. A data set comprising complexes that dissociate through the less common pathway was compared with a set of protein complexes that dissociate via subunit unfolding. Analysis of their subunit interfaces and charge densities suggests that complexes with fewer interfacial salt bridges and higher charge densities are more likely to undergo dissociation without unfolding. Overall, therefore, we show that while the relative strengths of interfacial interactions are the major determinants for the preferred disassembly routes in solution, subunit flexibility, charge density, salt bridges, and interface size influence dissociation in the gas phase.

## Experimental Procedures

### IM-MS Experiments

IM-MS experiments were performed on a quadrupole ion mobility time-of-flight mass spectrometer (Synapt HDMS, Waters Corp., Manchester, UK), which was modified so that the traveling-wave IM cell was replaced with an 18 cm drift cell with radial RF confinement (RF amplitude 200 V) and a linear voltage gradient along the axis of ion transmission (Bush et al., 2010). Two to three microliters of complex-containing solution (∼10 μM) was analyzed by nanoESI using gold-coated borosilicate capillaries prepared in-house. The following parameters were used: source pressure 4–6 mbar, capillary voltage 1.0–1.5 kV, sample cone voltage 20 V, bias voltage 20 V, IM entrance DC 5 V, trap gas 6 ml/min, trap collision energy 5 V. Helium (2 Torr) was used as the buffer gas, and the drift voltage varied from 50 to 200 V. For dissociation experiments, the trap collision energy was increased. Assignment of MS/MS spectra was aided by IM measurements. Peaks corresponding to nM^nz+^ and (n+1)M^(n+1)z+^ have identical *m/z* values, but experience different drift times through the mobility device (see Supplemental Experimental Procedures). Charge state averages, Z_*av,*_ were calculated by Gaussian fitting of the charge state envelope (Kaltashov and Mohimen, 2005) or multiple Gaussian distributions (Figures 1B, 2B, and 4B). An overall Z_*av*_ (Figure 5F) was calculated using Equation 1, where *I*_*i*_ is the intensity of the *i*th charge state, *Z*_*i*_ is the net charge on the *i*th charge state, and *N* is the total number of charge states observed (Iavarone et al., 2000).

(1) $$Z_{av}=\frac{\sum_{i=1}^{N}Z_{i}I_{i}}{\sum_{i=1}^{N}I_{i}}$$

### Preparation of Protein Complex Solutions

Human TTR, avidin from egg white, ADH from *Saccharomyces cerevisiae,* pyruvate kinase from rabbit heart, glutamate dehydrogenase from bovine liver, and β-galactosidase from *Escherichia coli* were obtained from Sigma Aldrich (St. Louis, MO, USA); human SAP was obtained from CalBioChem (Darmstadt, Germany). Tryptophan synthase (50 mM Bicine pH 7.8, 10 mM EDTA, 1 mM DTE, 0.02 mM pyridoxalphosphate) from *Salmonella enterica* was a gift from I. Schlichting, (Max Planck Institute, Heidelberg). NBDO from *Comamonas sp.* (50 mM bis-Tris pH 6.8, 5% glycerol, 1 mM DTT, trace ammonium sulfate) was provided by R. Friemann (University of Uppsala). MMOH from *Methylococcus capsulatus* and ToMOH from *Pseudomonas stutzeri* (25 mM MOPS, pH 7.0) were provided by S.J. Lippard (MIT). All proteins and complexes were buffer-exchanged into 200 mM AA, pH 7.0, using Micro Bio-Spin 6 columns (Bio-Rad, UK). AA buffer, TEA buffer TEA, 1, 8-diazabicycloundec-7-ene (DBU), and *m*-NBA were obtained from Sigma Aldrich (St. Louis, MO, USA). Charge reduction was carried out by the addition of TEA or DBU (10–20 mM). Supercharging was achieved by the addition of 1% *m*-NBA (final *v/v*).

### X-Ray Crystal Structure Calculations

PISA (Krissinel and Henrick, 2007) was used to calculate SASA, interface area, and the number of interfacial salt bridges. Salt bridges were permitted between basic (Lys, Arg) and acidic (Glu, Asp) residues, with a bond distance < 4 Å. *A*_rel_ values were calculated with Equation 2, where *A*_s_ is the SASA of the bound subunit in isolation from the rest of the complex, and *M* the monomer molecular mass. The *A*_rel_ of heteromers were averaged over each subunit type.

(2) $$A_{\text{rel}}=\frac{A_{\text{s}}}{4.84M^{0.76}}$$

The Student’s t test (one-sided) was used to determine statistical differences between atypically and typically dissociating complexes (two sample, unequal variance). CCS were calculated using the projection approximation (PA) method (Mack, 1925) implemented in MOBCAL (Mesleh et al., 1996; Shvartsburg and Jarrold, 1996). The PA underestimates experimental CCS, however, experimental CCS of globular complexes are highly correlated with PA CCS (Benesch and Ruotolo, 2011). A scaled PA value is reported (Equation 3), using an empirically determined scaling factor of 1.14 (root-mean-square deviation [rmsd] ± 3%) (Benesch and Ruotolo, 2011).

(3) $$CCS_{calc}=1.14CCS_{PA}$$

To calculate CCS of subunits or subassemblies, the corresponding atomic coordinates were manually removed from the coordinate file of the intact complex.

Solvent-free MD simulations of tryptophan synthase were carried out as described in the Supplemental Experimental Procedures.

## Supplemental Information

Document S1. Supplemental Discussion, Supplemental Experimental Procedures, Figures S1–S9, and Table S1
