# Supplementary material for: The Role of Salt Bridges, Charge Density, and Subunit Flexibility in Determining Disassembly Routes of Protein Complexes
Source: Structure. 2013 Aug 6;21(8):1325–37. doi: 10.1016/j.str.2013.06.004 (PMC3737473; doi:10.1016/j.str.2013.06.004)
Supplement: Document S1. Supplemental Discussion, Supplemental Experimental Procedures, Figures S1–S9, and Table S1 [file mmc1.pdf]

Structure, Volume *21*

Supplemental Information

The Role of Salt Bridges, Charge Density, and Subunit Flexibility in Determining Disassembly Routes of Protein Complexes

Zoe Hall, Helena Hernández, Joseph A. Marsh, Sarah A. Teichmann, and Carol V. Robinson

Inventory of Supplemental Information

Supplemental Data

Figure S1: Related to Figure 1, shows SAP dissociation is dependent on charge state and not additive

Figure S2: Related to Figure 2, shows the CCS of TTR CID products are not affected by the precursor ion charge state

Figure S3: Related to Figure 3, shows the CCS of higher oligomers of SAP and TTR formed by CID

Figure S4: Related to Figure 4, shows MS and CCS of intact tryptophan synthase

Figure S5: Related to Figure 5, shows changes in MS/MS spectra of tryptophan synthase with increasing precursor charge state

Figure S6: Related to Figure 6, shows MS spectra of complexes in buffer and in presence of *m*-NBA

Figure S7: Related to Figure 7, shows MS/MS spectra for avidin with increasing precursor ion charge state

Table S1: Related to Figure 6, compares MS-observed monomer charge states following CID with those expected from charge-symmetric dissociation

Supplemental Discussion

Supplemental Experimental Procedures

Supplemental References

Supplemental Data

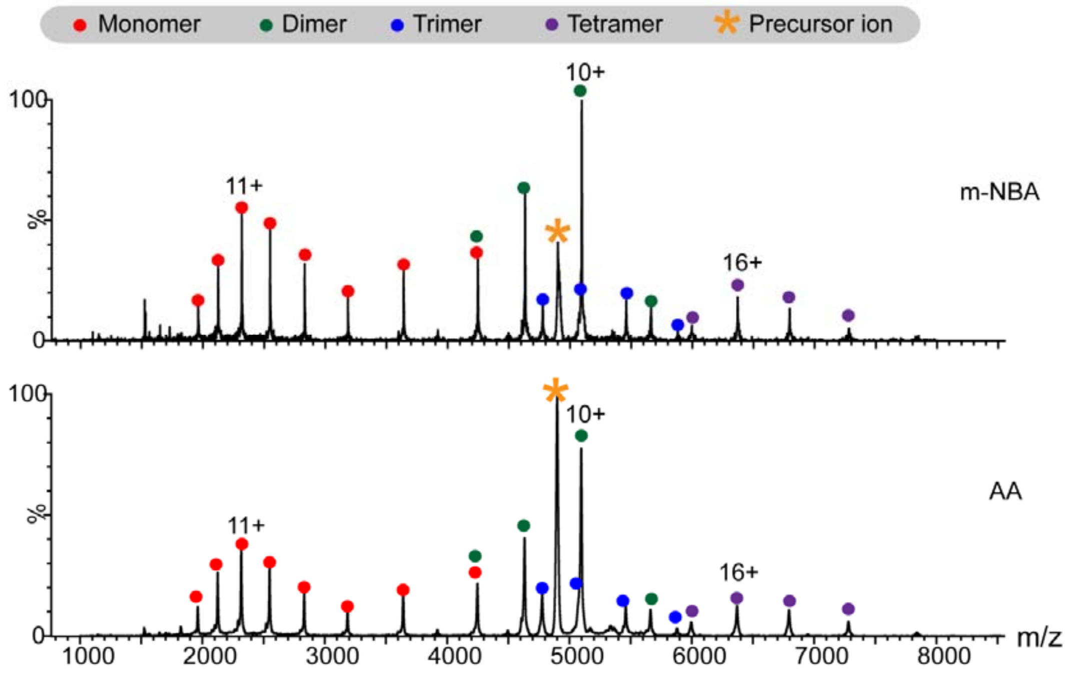

Figure S1. (Related to Figure 1) MS/MS of SAP 5-mer 26+ in the presence or absence of *m*-NBA. Similar dissociation patterns are observed for the 26+ ion, regardless of whether it was generated from AA buffered solutions, or from those solutions containing *m*-NBA.

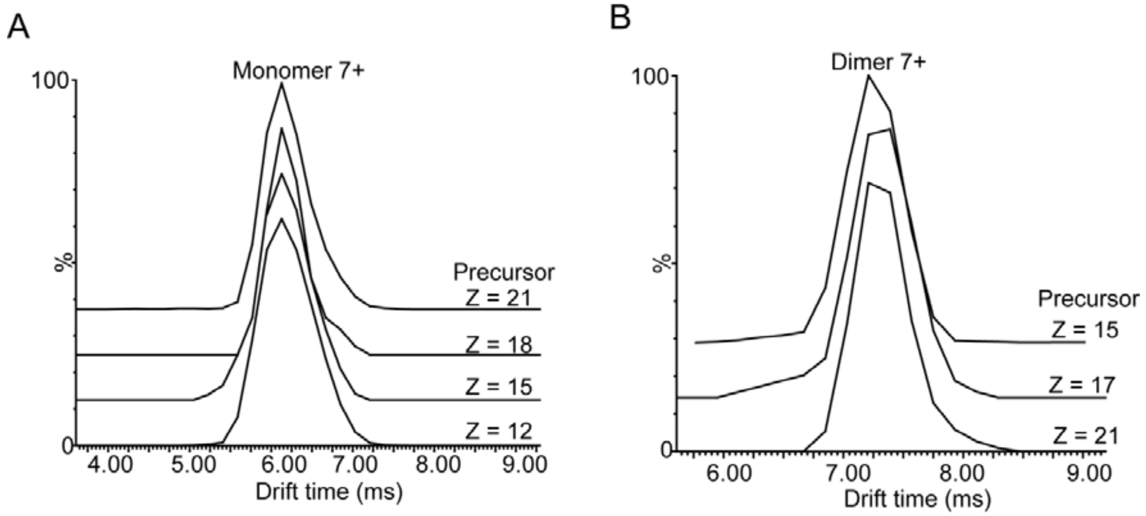

Figure S2. (Related to Figure 2) CCS of CID products are independent of the precursor ion charge state from which they arise. **A** and **B** show arrival time distributions for TTR monomer (7+) and dimer (7+), ejected from a range of precursor ion charge states.

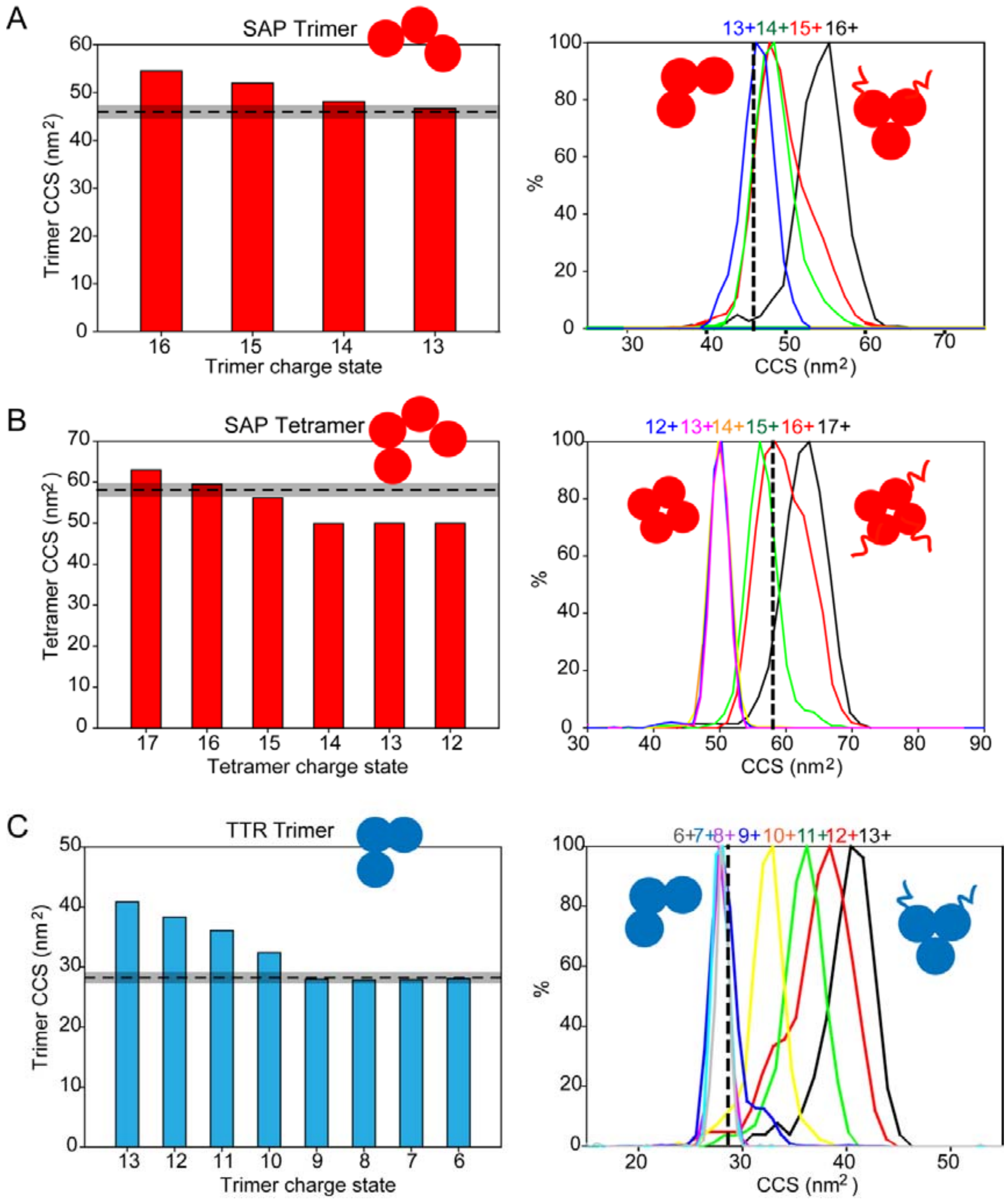

Figure S3. (Related to Figure 3) Higher order oligomers of SAP and TTR. CCS were measured for SAP 3-mer (**A**), SAP 4-mer (**B**) and TTR 3-mer (**C**) and compared with the CCS calculated for the corresponding X-ray crystal structures. CCS for each charge state is the average value calculated from multiple MS/MS experiments of different precursor ions which give the same charge product ions (standard deviation < 1 -2 %). Representative arrival time distributions for the individual charge states are shown on the right hand side. The dashed line and shaded region represents the calculated CCS ± 3 %. CCS for the lower charge states for SAP 4-mer suggest substantial collapse following the loss of a monomer.

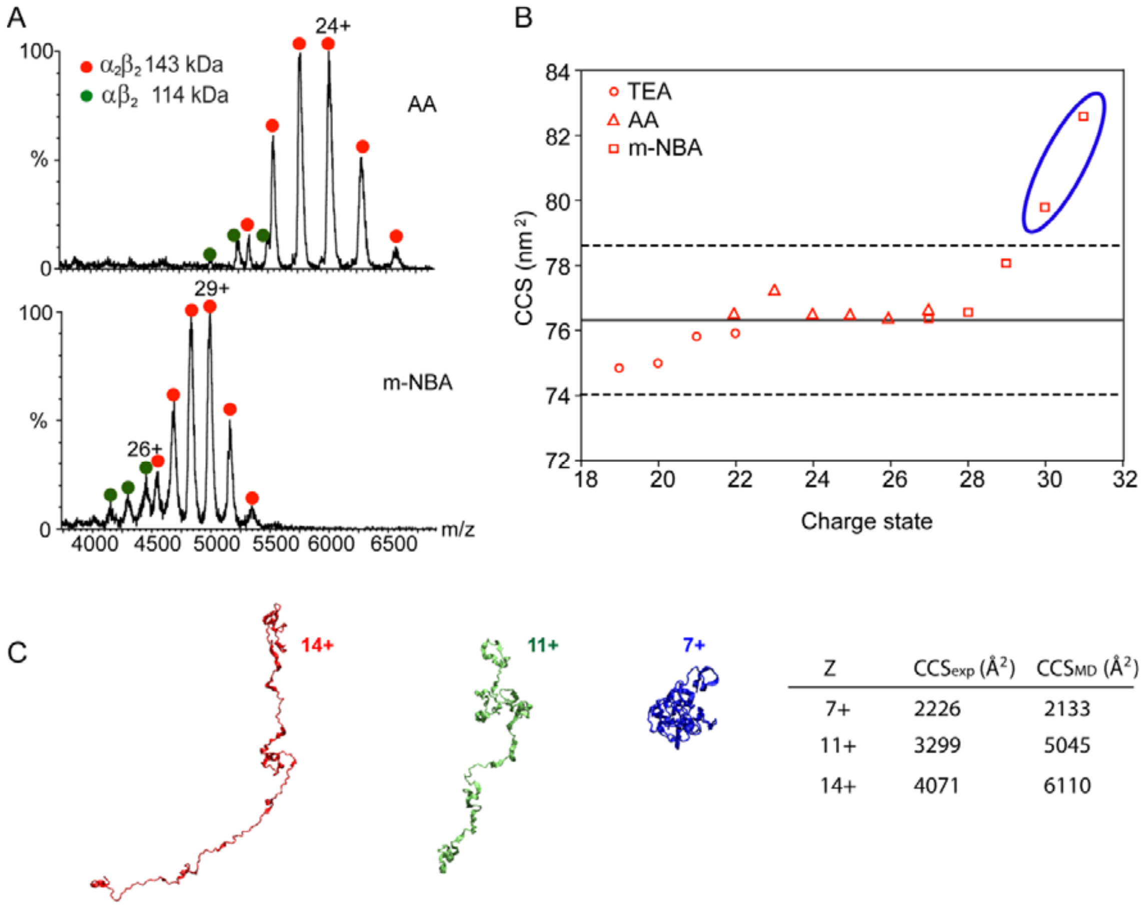

Z	CCSexp (Å²)	CCSMD (Å²)
7+	2226	2133
11+	3299	5045
14+	4071	6110

Figure S4. (Related to Figure 4) Charge manipulation of heteromeric tryptophan synthase. **A** MS spectra for tryptophan synthase in 200 mM AA, and with 1 % supercharging reagent *m*-NBA. A minor proportion of trimer is present in solution (green), in addition to the intact complex (red). **B** CCS of tryptophan synthase plotted against charge state. Data from TEA, AA and *m*-NBA-containing solutions are shown as circles, triangles and squares respectively. Unfolding is observed for charge states ≥ 30+. Literature CCS (charge-averaged) and associated error (± 3 %) are shown as solid and dashed lines (Hall, et al., 2012a). **C** Comparison of CCS for tryptophan synthase dissociated α-monomer from molecular dynamics and ion mobility experiments.

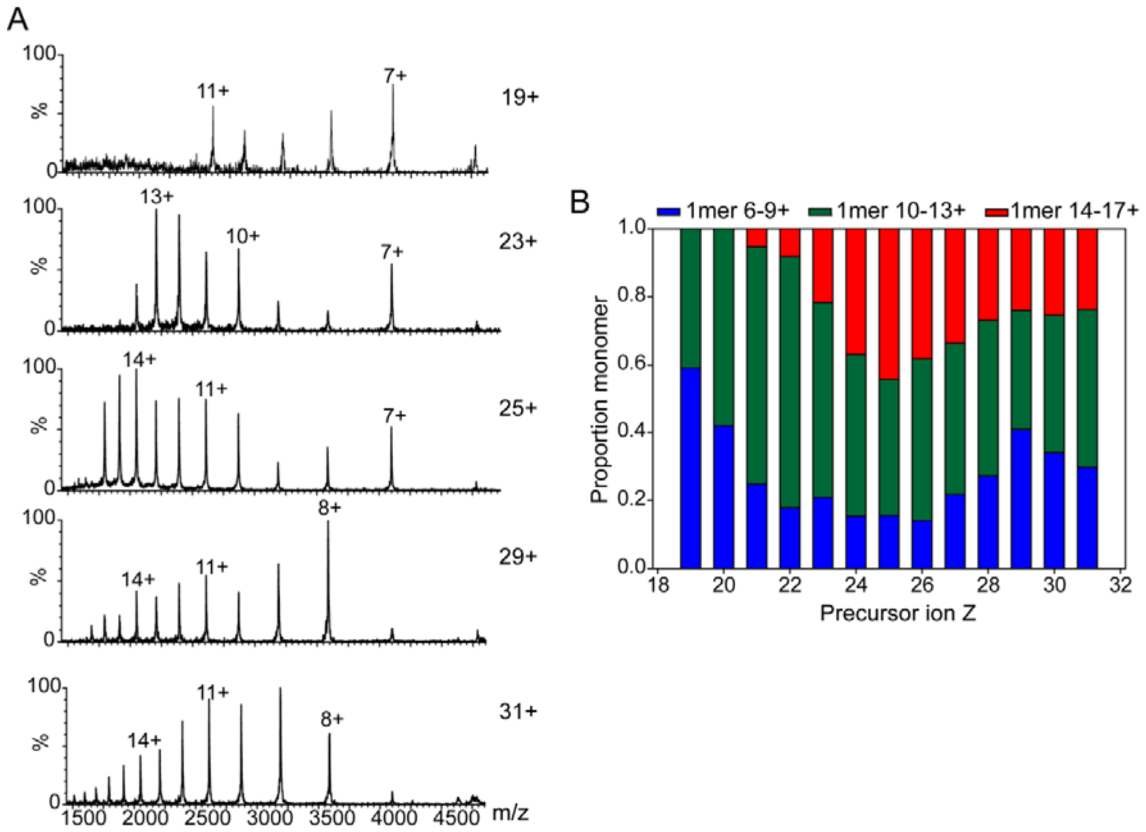

Figure S5. (Related to Figure 5) Charge-state dependent dissociation of tryptophan synthase. **A** MS/MS spectra showing the dissociated tryptophan synthase α-monomer with increasing precursor charge state, at similar laboratory frame energies. **B** The relative intensities of low (6-9+, blue), intermediate (10-13+, green) and high (14-17+, red) charged α-monomer are plotted against precursor ion charge state. Charge states were generated using the following additives to 200 mM AA: 19-21+ (20 mM TEA), 22-26+ (no additive), 27-31+ (1 % *m*-NBA).

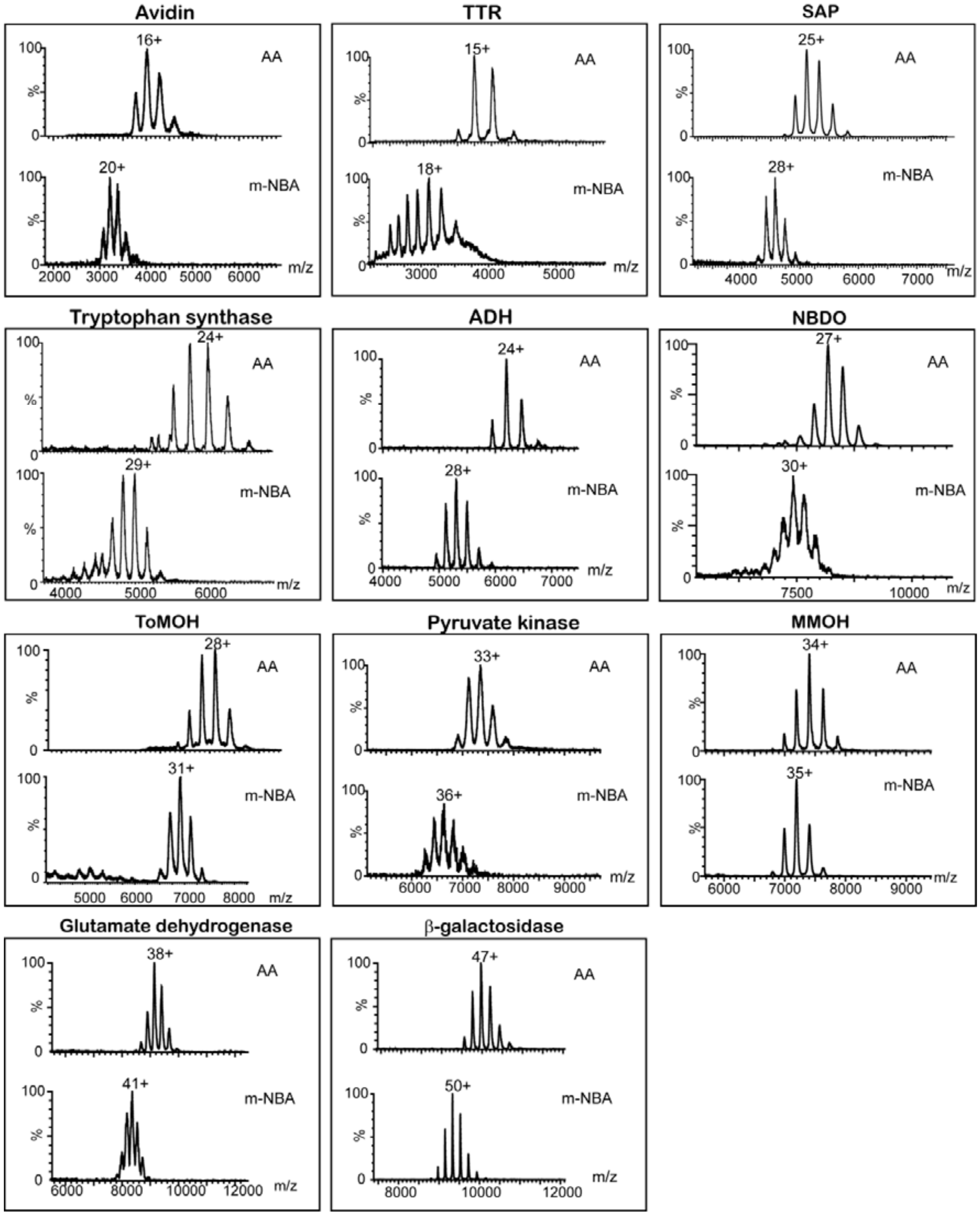

Figure S6. **(Related to Figure 6)** MS spectra of complexes in the presence of 200 mM AA (top spectrum in each panel) or 1 % *m*-NBA (bottom spectrum in panel).

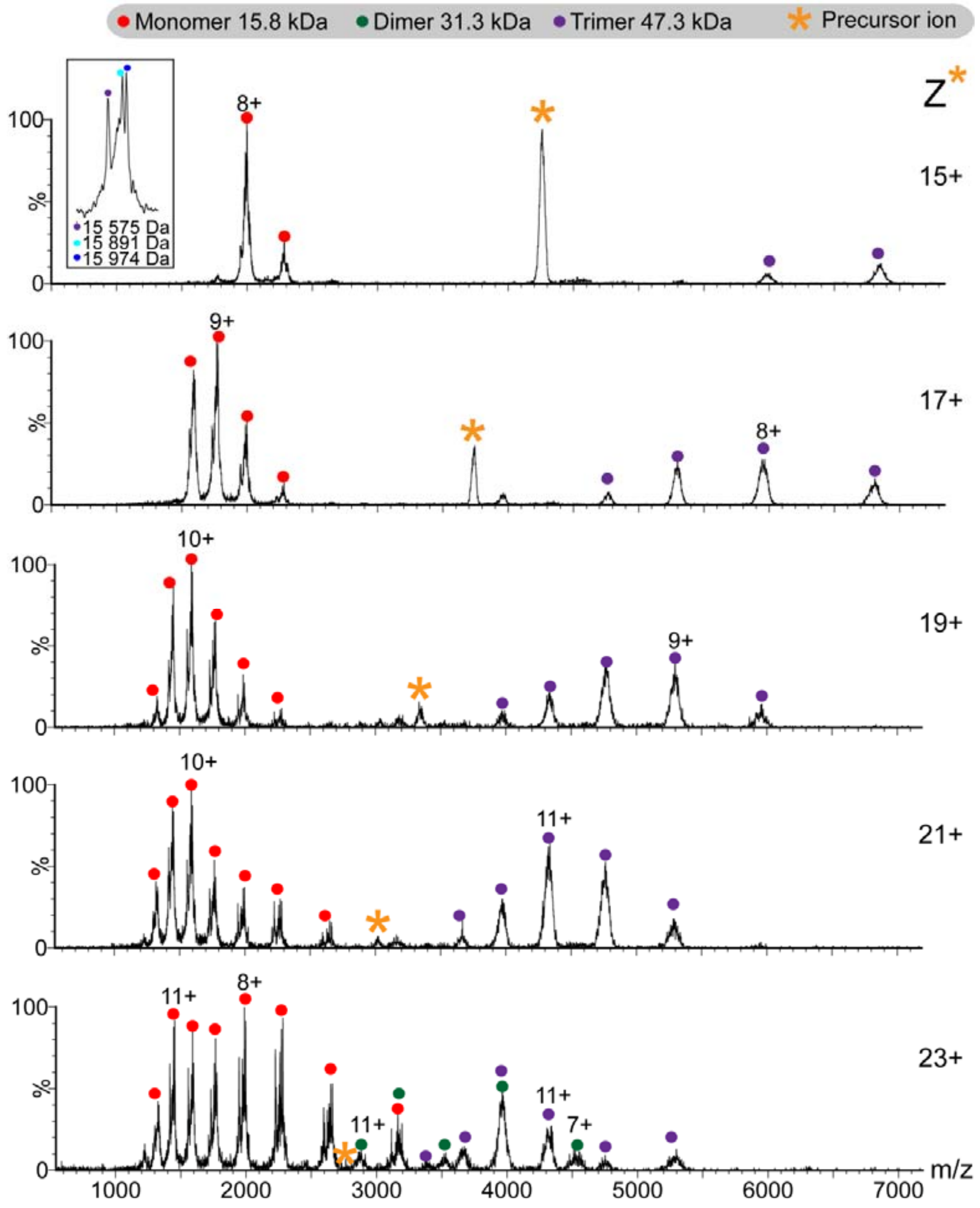

Figure S7. **(Related to Figure 7)**. MS/MS of avidin with increasing precursor ion charge state. Initially, the dissociated monomer charge state increases with the precursor ion charge state. As avidin charge state is increased further by the additional of supercharging *m*-NBA, avidin dissociates atypically, ejecting lower charged monomers and dimers.

Table S1. (Related to Fig. 6) The MS-observed monomer charge states following CID are compared with those expected from charge-symmetric dissociation. Additionally, complexes with lower solvent accessible surface area (SASA) experience a greater % increase in average charge state (Δ Z_{av}) in the presence of 1 % *m*-NBA supercharging reagent compared to that in ammonium acetate (AA).

Complex	SASA ($Å^2$)	Z_{av} 200mM AA	Z_{av} 1% *m*-NBA	[a]Δ Z_{av} (%)	[b]Symmetric 1-mer Z	[c]Observed 1-mer Z	
avidin 64 kDa, 4-mer	18190	15.8	19.2	21.5	4.8	5-12+ (16 kDa)	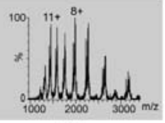
TTR 55 kDa, 4-mer	19290	14.6	18.8	28.8	4.3	6-12+ (14 kDa)	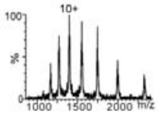
SAP 125 kDa, 5-mer	38190	24.6	28.2	14.6	5.6	6-14+ (25.5 kDa)	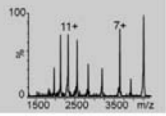
tryptophan synthase 143 kDa, $\alpha_2\beta_2$	43290	24.5	29.5	20.4	5.8	6-16+ (α 28 kDa)	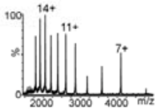
ADH 143 kDa, 4-mer	48930	23.8	27.8	16.8	7.0	16-21+ (37 kDa)	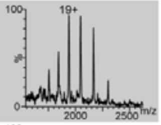
NBDO 218 kDa, $\alpha_3\beta_3$	58630	26.8	29.8	11.2	6.7	13-17+ (α 49 kDa)	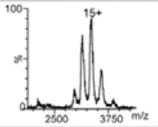
ToMOH 212 kDa, $\alpha_2\beta_2\gamma_2$	59750	28.4	31.0	9.2	1.5	4-9+ (γ 10 kDa)	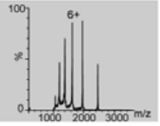
MMOH 251 kDa, $\alpha_2\beta_2\gamma_2$	60640	33.9	35.0	3.2	2.8	13-19+ (γ 20 kDa)	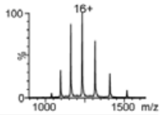
pyruvate kinase 232 kDa, 4-mer	69290	33.2	35.9	8.1	9.0	24-31+ (59 kDa)	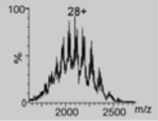
glutamate dehydrogenase 336 kDa, 6-mer	107290	38.4	40.6	5.7	6.8	25-32+ (56 kDa)	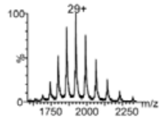
β-galactosidase 465 kDa, 4-mer	136712	47.2	49.9	5.7	12.5	[d]19-26+ (33 kDa)	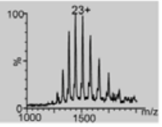

* Table legends ***a-d*** described overleaf

Table S1 legend

[a] % increase in MS-determined Z_{av} upon addition of 1% *m*-NBA.

[b] Expected monomer charge state if charge-symmetric dissociation occurs, e.g. ***n***-mer with ***Z*** charges ejects monomer with ***Z/n*** charges. Based on the dissociation of the intact complex in the presence of 1 % *m*-NBA.

[c] Range of charge states observed for monomer following CID of the intact complex in the presence of 1 % *m*-NBA.

[d] Monomer fragment (33.1 kDa) observed only. This may be the result of cleavage at Proline 7

Supplemental Discussion

It is worth considering the number of intrasubunit salt bridges present in the complexes dissociating atypically. A high number of intrasubunit salt bridges might inhibit gas-phase unfolding, and therefore predispose the complex towards atypical dissociation. In fact, complexes dissociating typically had a greater number of intrasubunit salt bridges. Whilst the number of intersubunit salt bridges are poorly correlated with interface area, there is a reasonable correlation ($R^2 = 0.8$) between the number of intrasubunit salt bridges and subunit area. Therefore the higher number of intrasubunit salt bridges in typically dissociating complexes is presumably due to their larger size on average, than the atypical complexes. No significant difference in intrasubunit salt bridge density (*i.e.* normalised for subunit area) was observed between the two groups of protein complexes.

It is also interesting to consider the experimentally observed gas-phase pathways for typically dissociating complexes, and for concanavalin A (3D4K) which loses a compact monomer, rather than the dimer predicted by the *in silico* disassembly. In all these cases (with the exception of HSP 16.9, which has unusually high subunit flexibility), the number of interfacial salt bridges broken in the experimentally observed gas-phase pathways were less than or equal to that predicted from the *in silico* disassembly pathway (Figure S8). This further supports the hypothesis that interfacial salt bridges are important for directing gas-phase disassembly routes.

It should be noted that two important assumptions have been made when considering the number of interfacial salt bridges broken during disassembly. The first is that the solution and gas phase structures are sufficiently similar, and the second is that salt bridges have not been neutralised during electrospray (ESI). Retention of native-like salt bridges in the gas phase, however has been shown previously (Breuker, et al., 2011), and it is likely that the interfacial salt bridges are protected from neutralisation during ESI, being buried in the interfaces and not exposed at surface.

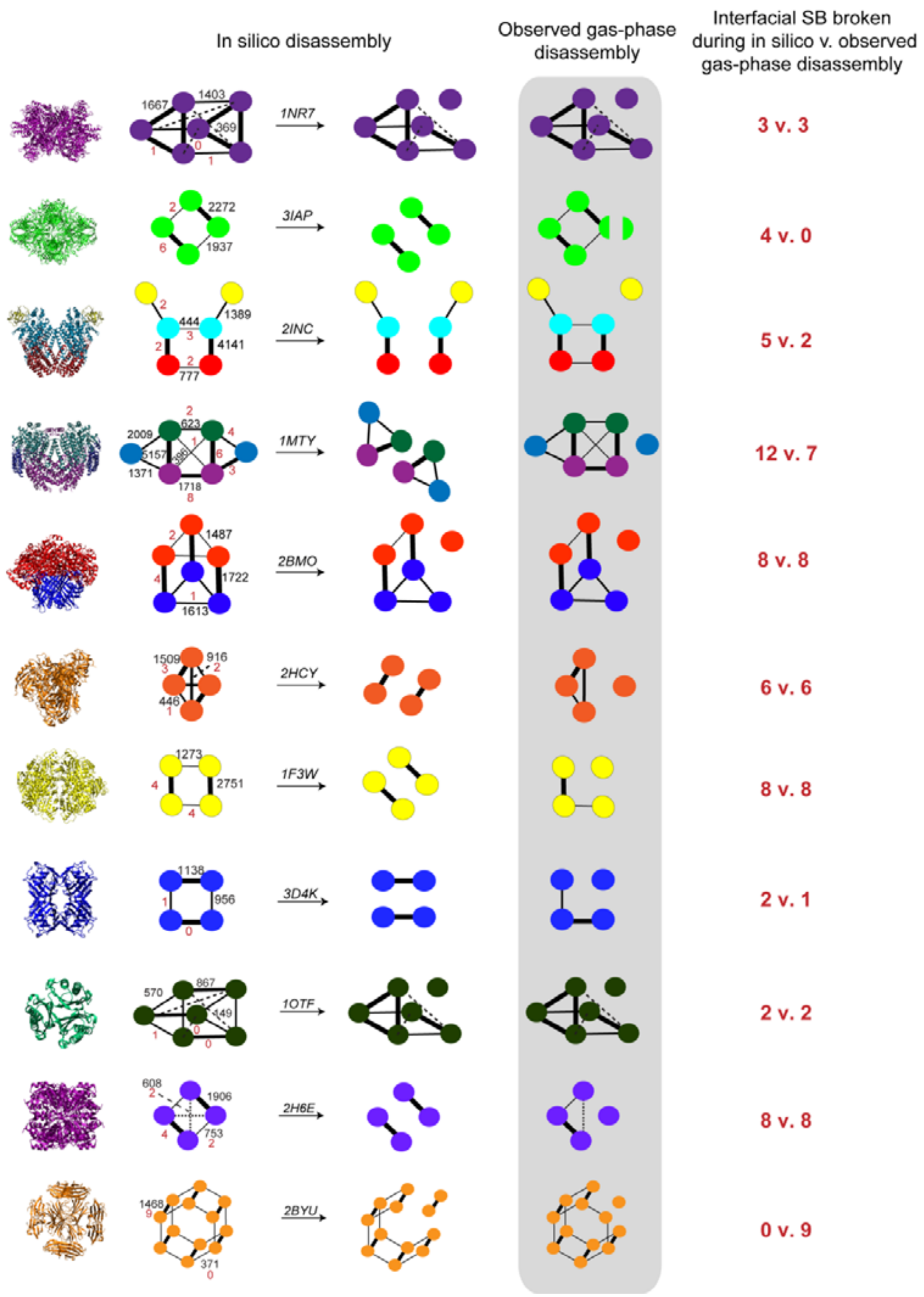

Figure S8. Comparing the number of interfacial salt bridges broken during *in silico* disassembly, with the number broken during experimentally observed gas-phase disassembly (highlighted grey). Complexes undergoing typical CID are shown, and concanavalin A (3D4K), which loses a compact monomer rather than the dimer predicted by the *in silico* disassembly.

An intriguing observation is that smaller complexes, which are more susceptible to undergo atypical CID, have higher charge densities on average than larger complexes. This is anticipated from the relationship between solvent accessible surface area (SASA) and Z_{av} as described previously (Kaltashov and Mohimen, 2005), and highlights the importance of charge in promoting atypical dissociation (Figure S9A,B). Furthermore, smaller complexes tend to have a greater % increase in Z_{av} when exposed to a given concentration of *m*-NBA (Table S1, Figure S9C) than larger complexes. This may be related to the fact that smaller complexes have a greater surface area to volume ratio. This has implications for the mechanism of supercharging. Whilst charge density can be considered to be a driving force behind atypical *versus* typical dissociation, it is the combination of accessible surface area and supercharging reagent concentration that determines the charge density. Based upon this, a higher concentration of supercharging reagent might be expected to induce atypical dissociation in some of the typically dissociating complexes, however high concentrations of supercharging reagent may also increase unfolding in solution (Figure S9D).

The ability to manipulate dissociation pathways in the gas phase by altering the charge state is an intriguing prospect. However, the potential for unfolding of the intact complex during electrospray, particularly in the presence of supercharging reagents, is one serious limitation that has already been proposed (Sterling, et al., 2011; Sterling, et al., 2012). Evidence for this was observed for the higher charge states of tryptophan synthase (Figure S4). Increasing the charge state without unfolding is therefore a major objective. One attractive option could be the use of cross-linking strategies to hinder unfolding, as used in early investigations into the origin of the asymmetric charge partitioning in CID (Jurchen and Williams, 2003). Further development of alternative supercharging strategies that do not unfold complexes would also be of major significance. Despite evidence for unfolding of the intact complex, supercharging can also promote atypical dissociation of folded subunits, implying that these pathways are independent and not mutually exclusive (Zhou, et al., 2013).

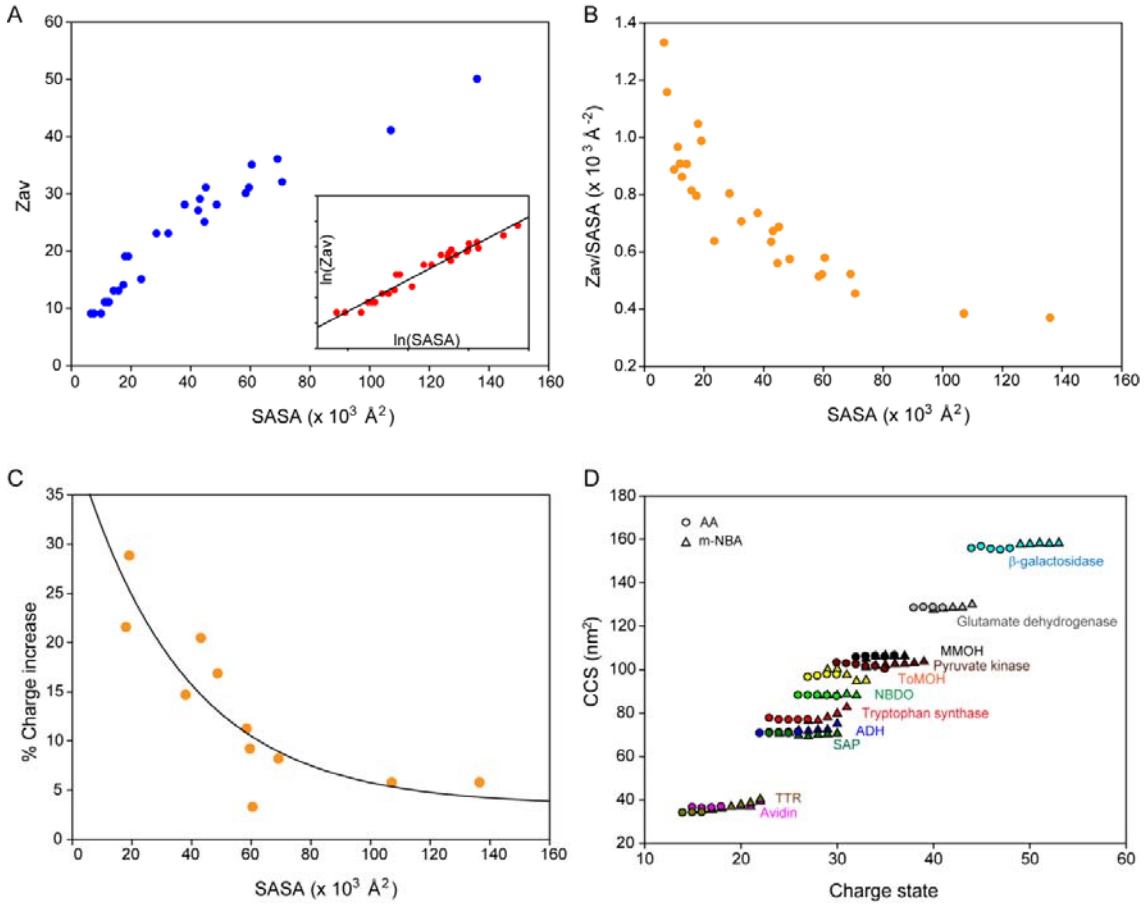

Figure S9. Relationship between complex size, as described by SASA, and Z_{av} (**A**) (all complexes in Figure 6). As the size of the complex decreases, the charge density (Z_{av}/SASA) increases exponentially (**B**). Increase in MS-observed Z_{av} upon addition of 1 % *m*-NBA, plotted as a function of complex SASA (complexes in this study only). (**C**). CCS of parent complex ion in the presence of AA (circles) or 1% *m*-NBA (triangles) plotted as a function of charge state (**D**). An increase in CCS consistent with unfolding is observed for the highest charge states of several complexes.

Supplemental Experimental Procedures

Ion mobility aids charge state assignment in MS/MS spectra. Peaks corresponding to nM^{nz+} and $(n+1)M^{(n+1)z+}$ have identical *m/z* values, however typically experience different IM drift times, as shown below for MS/MS spectra of SAP 26+ (**A**) and TTR 21+ (**B).**

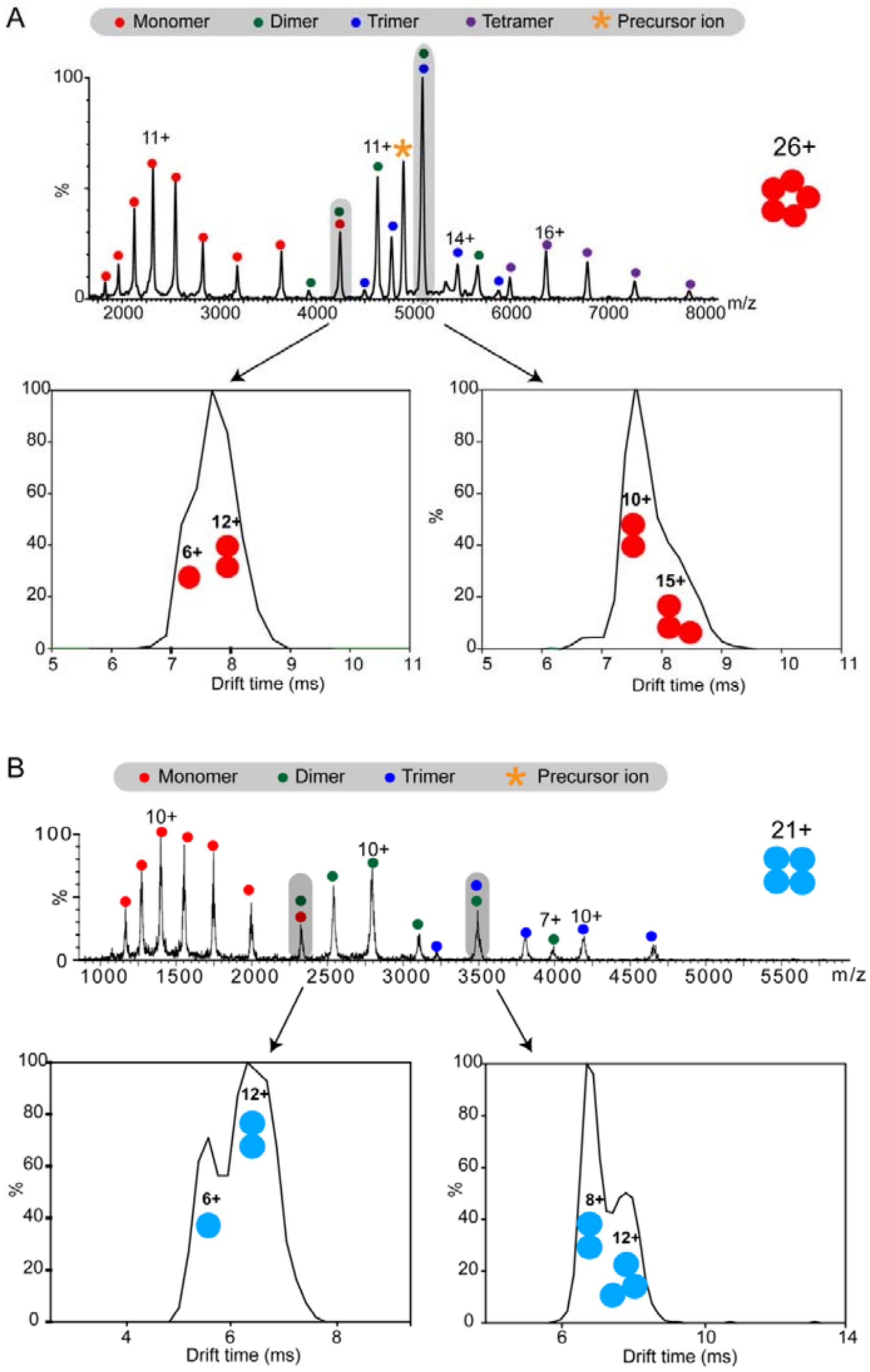

Molecular dynamics simulations

Simulations were run in double floating-point precision, with GROMACS (van der Spoel, et al., 2005) 4.5.3. Tetrameric tryptophan synthase (PDB 1WBJ) was simulated in a solvent-free system using the OPLS-AA/L forcefield (Kaminski, et al., 2001). We based our simulation method on previously published procedure (Hall, et al., 2012b; Pagel, et al., 2013; Patriksson, et al., 2007) for simulating proteins *in vacuo*. A steepest descent energy minimisation was performed, followed by 1 ns simulations, with randomly generated initial velocities. Neither periodicity nor cut-offs were employed in the calculations. Energy conservation was achieved using a 1 fs integration step, and constraining bonds to hydrogen with the LINCS algorithm (Hess, et al., 1997). Simulations of tryptophan synthase 24+ were carried out over a linear temperature gradient (300 - 1000 K) in order to induce dissociation. Different charge configurations were assigned to the starting structure, such that one α-monomer had 14, 11, or 7 charges, as determined by MS/MS experiments. The remaining charges were distributed evenly over the residual accessible basic residues (Hall, et al., 2012b). These 'charge isomers' were used as different seeding structures for simulations, to evaluate the importance of the specific arrangement of charges. In order to identify the most populated conformations, an RMSD-clustering algorithm (Daura, et al., 1999) was used.

Supplemental References

Breuker, K., Brüschweiler, S., and Tollinger, M. (2011). Electrostatic stabilization of a native protein structure in the gas phase. Angew. Chem. Int. Ed. *50*, 873-877.

Daura, X., Gademann, K., Jaun, B., Seebach, D., van Gunsteren, W.F., and Mark, A.E. (1999). Peptide folding: When simulation meets experiment. Angew. Chem. Int. Ed. *38*, 236-240.

Hall, Z., Politis, A., and Robinson, C.V. (2012a). Structural modeling of heteromeric protein complexes from disassembly pathways and ion mobility-mass spectrometry. Structure *20*, 1596-1609.

Hall, Z., Politis, A., Bush, M.F., Smith, L.J., and Robinson, C.V. (2012b). Charge-state dependent compaction and dissociation of protein complexes: Insights from ion mobility and molecular dynamics. J. Am. Chem. Soc. *134*, 3429-3438.

Hess, B., Bekker, H., Berendsen, H.J.C., and Fraaije, J.G.E.M. (1997). LINCS: A linear constraint solver for molecular simulations. J. Comput. Chem. *18*, 1463-1472.

Jurchen, J.C., and Williams, E.R. (2003). Origin of asymmetric charge partitioning in the dissociation of gas-phase protein homodimers. J. Am. Chem. Soc. *125*, 2817-2826.

Kaminski, G.A., Friesner, R.A., Tirado-Rives, J., and Jorgensen, W.L. (2001). Evaluation and reparametrization of the OPLS-AA force field for proteins via comparison with accurate quantum chemical calculations on peptides. J. Phys. Chem. B *105*, 6474-6487.

Kaltashov, I.A., and Mohimen, A. (2005). Estimates of protein surface areas in solution by electrospray ionisation mass spectrometry. Anal. Chem. *77*, 5370-5379.

Pagel, K., Natan, E., Hall, Z., Fersht, A.R., and Robinson, C.V. (2012). Intrinsically disordered p53 and its complexes populate compact conformations in the gas phase. Angew. Chem. Int. Ed. *52*, 361-365.

Patriksson, A., Marklund, E., and van der Spoel, D. (2007). Protein structures under electrospray conditions. Biochemistry *46*, 933-945.

Sterling, H.J., Cassou, C.A., Trnka, M.J., Burlingame, A.L., Krantz, B.A., and Williams, E.R. (2011). The role of conformational flexibility on protein supercharging in native electrospray ionization. Phys. Chem. Chem. Phys. *13*, 18288-18296.

Sterling, H., Kintzer, A., Feld, G., Cassou, C., Krantz, B., and Williams, E. (2012). Supercharging protein complexes from aqueous solution disrupts their native conformations. J. Am. Soc. Mass Spectrom. *23*, 191-200.

van der Spoel, D., Lindahl, E., Hess, B., Groenhof, G., Mark, A.E., and Berendsen, H.J.C. (2005). GROMACS: Fast, flexible, and free. J. Comput. Chem. *26*, 1701-1718.

Zhou, M., Dagan, S., and Wysocki, V.H. (2013). Impact of charge state on gas-phase behaviors of noncovalent protein complexes in collision induced dissociation and surface induced dissociation. Analyst. *138*, 1353-1362.
